# FABP4 is an independent risk factor for lymph node metastasis and poor prognosis in patients with cervical cancer

**DOI:** 10.1186/s12935-021-02273-4

**Published:** 2021-10-26

**Authors:** Guoqing Li, Qiulei Wu, Lanqing Gong, Xiaohan Xu, Jing Cai, Linjuan Xu, Ya Zeng, Xiaoqi He, Zehua Wang

**Affiliations:** Department of Obstetrics and Gynecology, Union Hospital, Tongji Medical College, Huazhong University of Science and Technology, Wuhan, 430022 China

**Keywords:** Cervical cancer, Lymph node metastasis, Prognosis, FABP4

## Abstract

**Background:**

Pelvic lymph node metastasis (LNM) is a crucial independent prognostic factor in cervical cancer (CCa) and serves as an indicator for radiation therapy as the primary or an adjuvant treatment option. However, preoperative diagnosis of LNM remains challenging. Thus, we aimed to identify biomarkers of LNM in patients with presumed early-stage CCa.

**Methods:**

The differentially expressed genes (DEGs) between tumours with different lymph node statuses were identified by using The Cancer Genome Atlas database. Then, univariate Cox regression analysis and Kaplan–Meier analyses were utilized to screen overall survival (OS)-associated genes. Multivariate Cox analysis and logistical analysis were utilized to evaluate independent risk factors for OS and LNM, respectively. Subsequently, the protein level of fatty acid binding protein 4 (FABP4) was detected in normal cervical and CCa tissues by immunohistochemistry assays. EdU assays were performed to determine whether FABP4 altered the proliferation of cervical cancer cells. Wound healing and Transwell assays were conducted to explore the effects of FABP4 depletion on migratory and invasive abilities of cervical cancer cells. F-actin fluorescence staining were performed to investigate morphological change and Western blotting analyses were performed to determine epithelial mesenchymal transition-related marker expression and downstream signalling pathways.

**Results:**

A total of 243 DEGs, including 55 upregulated and 188 downregulated DEGs, were found in CCa patients with LNM versus those without LNM. Among these, FABP4 was found to be closely associated with poor OS. Multivariate analysis uncovered that FABP4 was an independent risk factor for OS and LNM in patients with CCa. The immunohistochemical results verified dramatically increased FABP4 expression in CCa tissues compared to normal cervical epithelia and its association with poor OS and LNM. In vitro, The proliferation, migration and invasion of cervical cancer cells were significantly inhibited after knocking down of FABP4, which was accompanied by elevated expression of E-cadherin and downregulated expression of N-cadherin, Vimentin and p-AKT.

**Conclusions:**

FABP4 might be a promising biomarker of LNM and survival in patients with early-stage CCa and therefore could significantly contribute to the development of personalized prognosis prediction and therapy optimization.

**Supplementary Information:**

The online version contains supplementary material available at 10.1186/s12935-021-02273-4.

## Background

Cervical cancer (CCa) is the second most prevailingly diagnosed cancer and the leading cause of cancer-related death among women in less developed countries [1, 2]. Pelvic lymph node metastasis (LNM) is a crucial independent prognostic factor in CCa, and patients with positive lymph nodes have a higher risk of recurrence and shorter 5-year survival rate [3–5]. In 2018, the International Federation of Gynecology and Obstetrics (FIGO) released revised staging guidelines that included lymph node status in the staging system, which assigns patients with positive pelvic lymph nodes as stage IIIC1 and those with para-aortic lymph nodes as stage IIIC2 [6]. Several researches have shown that radical hysterectomy plus chemoradiation does not prolong survival time in patients with positive lymph nodes compared with definitive chemoradiation alone [7–9]. The European Society of Gynecological Oncology guidelines recommend that lymph node status should be determined preoperatively or intraoperatively as much as possible to assess the prognosis and determine treatment strategy [10]. Therefore, it is urgently needed for patients with CCa to explore a technical method for early assessment of lymph node status.

Our previous study found that 14.6% of patients with early-stage CCa had LNM before surgery [11]. However, traditional imaging techniques are not sufficiently reliable to detect LNM (in a meta-analysis, the pooled sensitivity was 0.57 for computed tomography, 0.66 for positron emission tomography and 0.54 for magnetic resonance imaging) [12]. Moreover, no effective molecular markers have been identified for predicting LNM. Thus, the identification of novel and promising biomarkers associated with LNM is an urgency for patients with early-stage CCa.

FABP4, also known as A-FABP, is an intracellular lipid chaperone that can reversibly bind hydrophobic ligands, and has the ability to carry fatty acids to several organelles [13]. Numerous studies have shown that FABP4 plays a malignant role in metastatic cancers, such as colon cancer [14], breast cancer [15] and ovarian cancer [16]*.* However, the relationship between FABP4 and LNM of CCa has few been reported. Herein, we used bioinformatics methods to determine that fatty acid binding protein 4 (FABP4) is associated with LNM and the poor prognosis of patients with CCa, which was further confirmed through clinical data and experimental models, suggesting that FABP4 could be a potential biomarker of evaluating lymph node status and prognosis in CCa.

## Methods

### Data acquisition and processing

RNA-Seq profiles and clinical information from CCa patients were downloaded from The Cancer Genome Atlas (TCGA) database (available at: https://portal.gdc.cancer.gov/) to identify the key genes associated with LNM and prognosis. To ensure detection reliability, genes with FPKM values less than 0.5 in more than 50% of the samples were removed from further analysis. Samples meeting the following criteria were used for subsequent analysis: (1) samples with complete survival information, (2) follow-up time > 90 days, and (3) LNM information without missing values. The characteristics of the patients are summarized in Table 1. In addition, GSE15166 and GSE7410 from the Gene Expression Omnibus (GEO) database (available at: https://www.ncbi.nlm.nih.gov/geo/) were downloaded to validate the association between the expression level of FABP4 and clinicopathological features.

**Table 1:** Clinicopathological characteristics of patients in TCGA cohort (N = 178)

Variables	Number of patients (%)	Patient with LNM (%)
Age (years)		
Median (range)	46 (21–80)	
< 50	114 (64.0)	38 (58.5)
≥ 50	64 (36.0)	27 (41.5)
FIGO stage		
Stage I	109 (61.2)	24 (36.9)
Stage II	31 (17.4)	7 (10.8)
Stage III	16 (9.0)	14 (21.5)
Stage IV	20 (11.2)	18 (27.7)
Unknown	2 (1.1)	2 (3.1)
Histological type		
Squamous cell carcinoma	144 (80.9)	56 (86.2)
Adenocarcinoma	34 (19.1)	9 (13.8)
T stage		
T1	113 (63.5)	31 (47.7)
T2	38 (21.3)	15 (23.1)
T3	6 (3.4)	5 (7.7)
T4	14 (7.9)	9 (13.8)
Unknown	7 (3.9)	5 (7.7)
M stage		
M0	91 (51.1)	22 (33.8)
M1	13 (7.3)	11 (16.9)
Unknown	74 (41.6)	32 (49.2)

*LNM* lymph node metastasis, *FIGO* International Federation of Gynecology and Obstetrics

### Identification of differentially expressed genes (DEGs)

To identify LNM-associated genes in CCa, DESeq2 was used to screen the DEGs between patients with LNM and without LNM, and a *P*-value < 0.05 and the absolute value of logFC > 1 were set as the cut-off criteria.

### Functional enrichment analysis

To reveal the function of the 243 LNM-associated genes, Gene Oncology (GO) and Kyoto Encyclopedia of Genes and Genomes (KEGG) enrichment were analysed using the clusterProfiler package. To analyse the hallmark gene sets involved in FABP4, we downloaded the hallmark gene sets from the Molecular Signatures Database (available at: http://www.gsea-msigdb.org/gsea/msigdb/index.jsp) and performed gene set enrichment analysis (GSEA) between the FABP4 highly expressed and FABP4 low expressed groups in the TCGA cohort [17]. GSEA was also performed in GSE15166 and GSE7410 datasets. A *P*-value < 0.05 was considered statistically significant.

### Tissues samples and Immunohistochemistry (IHC)

Forty-eight normal cervical tissues of patients with uterine myoma who underwent hysterectomy and sixty-seven CCa samples from patients who did not undergo chemotherapy or radiotherapy were obtained from surgical resections for IHC analysis. All patient samples were collected at Union Hospital, Tongji Medical College, Huazhong University of Science and Technology, and written informed consent was obtained before surgery. All procedures related to the clinical samples were approved by the Ethics Committee of Tongji Medical College, Huazhong University of Science and Technology (IORG No: IORG0003571). The clinical characteristics of the patients are summarized in Table 2. A standard IHC protocol was performed to detect the protein level of FABP4. Briefly, formalin-fixed, paraffin-embedded normal cervical tissues and CCa tissues were deparaffinized and hydrated by sequential washing with xylene, anhydrous ethanol, 95% ethanol, 75% ethanol, 50% ethanol and PBS. After antigen retrieval, hydrogen peroxide solution was used to inactivate endogenous peroxidase. Nonspecific binding sites were subsequently blocked with 10% goat serum. The sections were incubated overnight with anti-FABP4 antibody (1:50 dilution; Proteintech, 12802-1-AP, China) at 4 °C. Subsequently, the sections were incubated with secondary antibody at 37 °C for 20 min followed by 3,3′-diaminobenzidine staining and counterstained with haematoxylin. The IHC results were scored according to the following formula: staining intensity (none = 0, weak = 1, moderate = 2, strong = 3) and staining area (none = 0, less than 30% = 1, between 30 and 60% = 2, more than 60% = 3).

**Table 2:** Clinicopathological characteristics of patients with CCa in our hospital (N = 67)

Variables	Number of patients (%)	Patient with LNM (%)
Age (years)		
Median (range)	45 (28–62)	
< 50	47 (70.1)	10 (66.7)
≥ 50	20 (29.9)	5 (33.3)
FIGO stage		
Stage I	45 (67.2)	9 (60.0)
Stage II	22 (32.8)	6 (40.0)
Histological type		
Squamous cell carcinoma	50 (74.6)	13 (86.7)
Adenocarcinoma	15 (22.4)	2 (13.3)
Others	2 (3.0)	0 (0.0)
Grade		
G1	14 (20.9)	0 (0.0)
G2	29 (43.3)	7 (46.7)
G3	19 (28.4)	6 (40.0)
Unknown	5 (7.5)	2 (13.3)
LVSI		
Absent	58 (86.6)	9 (60.0)
Present	9 (13.4)	6 (40.0)

*CCa* cervical cancer, *LNM* lymph node metastasis, *FIGO* International Federation of Gynecology and Obstetrics, *LVSI* lymphovascular space involvement

### Cell culture and transfection

Human cervical cancer cell lines (HeLa, SiHa, Caski) were purchased from the China Center for Type Culture Collection (Wuhan University, Wuhan, China) and cultured in RPMI-1640 media containing 10% foetal bovine serum (Gibco, USA). Among them, HeLa is a human cervical adenocarcinoma cell line, SiHa is a human cervical squamous cell carcinoma cell line, Caski is a human cervical carcinoma intestinal metastasis cell line. All cells were authenticated by short tandem repeat profiling and maintained in a humidified atmosphere at 37 °C and 5% CO_2_. FABP4 small interfering RNAs (siRNAs) were purchased from RiboBio (Guangzhou, China), and transfection was performed using Lipofectamine 3000 (Invitrogen, USA) according to the manufacturers’ protocol. The target sequences for the siRNAs were FABP4 siRNA #1 (CACGAGAGTTTATGAGAGA), FABP4 siRNA #2 (GGCATGGCCAAACCTAACA), and FABP4 siRNA #3 (GGAAAATCAACCACCATAA).

### RNA extraction and quantitative Real-Time PCR (qRT-PCR)

qRT-PCR were performed to test interfering efficiency of siRNAs. In brief, total RNAs of transfected cells were isolated using TRIzol reagent (Takara, Japan), followed by reverse transcription. qRT-PCR was performed with SYBR Green PCR Master Mix (Vazyme, China) on a Step-One Plus Real-Time PCR System. The relative expression of each mRNA was normalized using 2^−∆∆Ct^ method. Each experiment was performed in triplicate. The primers used in this study are β-actin (Forward): 5 ‘-CATGTACGTTGCTATCCAGGC-3’, β-actin (Reverse): 5 ‘-CTCCTTAATGTCACGCACGAT-3’, FABP4 (Forward): 5 ‘-ACAGGAAAGTCAAGAGCACCA-3’, FABP4 (Reverse): 5 ‘-TGGTGGTTGATTTTCCATCCCA-3’.

### EdU cell proliferation assay

EdU assay (RiboBio, Guangzhou, China) was performed to detect the effect of FABP4 on the proliferation of cervical cancer cells. Briefly, transfected cells were plated in 96-well plates at 1 × 10^4^ per well and incubated with 50 μM EdU for 2 h. Subsequently, the wells were washed with PBS and fixed with 4% paraformaldehyde followed by Apollo staining and Hoechst33342 staining. The ratio of the number of EdU-positive nuclei and Hoechst-stained nuclei represents the cell proliferation rate. The experiment was performed in three biological replicates.

### Wound healing and Transwell assay

For the wound healing assay, cells were plated in six-well plates at 3 × 10^5^ cells per well. After transfection with siRNAs, the cells were cultured for 48 h. When the cells formed a confluent monolayer, they were scratched with a 200 μL pipette tip. The wound areas were photographed with an inverted light microscope (Olympus, Japan) at 0 h and 24 h. The proportions of wound healing areas were measured using ImageJ (version 1.51) software and represented the migratory properties of cancer cells. For the Transwell assay, 200 μL of serum-free media containing 5 × 10^4^ cells was plated on the upper surface of chambers that were coated without (migration assay) or with (invasion assay) Matrigel, and 600 mL complete media was added to the bottom chambers. After 24 h of incubation at 37 °C, 4% paraformaldehyde was utilized to fix the cells that cross the 8 μm pore membranes. Then, the cells were stained with 0.1% crystal violet. The number of cells in five random visual fields of each insert was counted to evaluate the migratory and invasive properties of cancer cells at 200 × magnification. Each experiment was performed three times.

### F-actin fluorescence staining

To visualize the cytoskeleton of cervical cancer cells, F-actin was stained with TRITC phalloidin (Yeasen, Shanghai, China). The transfected cells were fixed with 4% formaldehyde and washed with PBS (pH 7.4) twice for 5 min each time. Then, TRITC phalloidin containing 1% bovine albumin was added and the reaction took place for 30 min. Subsequently, the cell nuclei were stained with DAPI. The cell morphology was imaged under a fluorescence microscope (Olympus, Japan).

### Western blotting analysis

The protein levels of E-cadherin, N-cadherin, Vimentin, FABP4, AKT, p-AKT, MAPK, p-MAPK and β-actin in cervical cancer cells were detected by Western blotting. The primary antibodies used were anti-E-cadherin (1:10,000 dilution; Abcam, ab76319, USA), anti-N-cadherin (1:2000 dilution; Proteintech, 66219-1-Ig, China), anti-Vimentin (1:1000 dilution; Abcam, ab92547, USA), anti-FABP4 (1:1000 dilution; Proteintech, 12802-1-AP, China), anti-AKT (1:2000 dilution; CST, 2920S, USA), anti-p-AKT (1:2000 dilution; CST, 4060S, USA), anti-MAPK (1:1000 dilution; CST, 4695S, USA), anti-p-MAPK (1:2000 dilution; CST, 4370S, USA) and anti-β-actin (1:5000 dilution; Proteintech, 66009-1-Ig, China). Iamge J software was applied to quantified the results of western blotting. Each experiment was performed in three biological replicates.

### Statistical analysis

All statistical analyses were performed using the R environment (version 3.6.3) and GraphPad Prism (version 8.0.2). Kaplan–Meier (KM) analyses for overall survival (OS, time from initial diagnosis to death or end of follow-up) were performed using survival and survminer packages, and the log-rank test was utilized. Univariate and multivariate Cox regression analyses were performed to screen survival-associated genes by using the survival package. Pearson correlation analysis was performed to determine the relationship of survival-associated genes. Binary logistical regression analysis was applied to test the risk for LNM in CCa. The association between FABP4 and clinicopathological characteristics was evaluated using the Mann–Whitney *U* test. Significant differences between different groups in cellular experiments were evaluated by Student’s *t* test. A *P*-value < 0.05 was considered statistically significant.

## Results

### Identification of LNM-associated DEGs

**Fig. 1:**
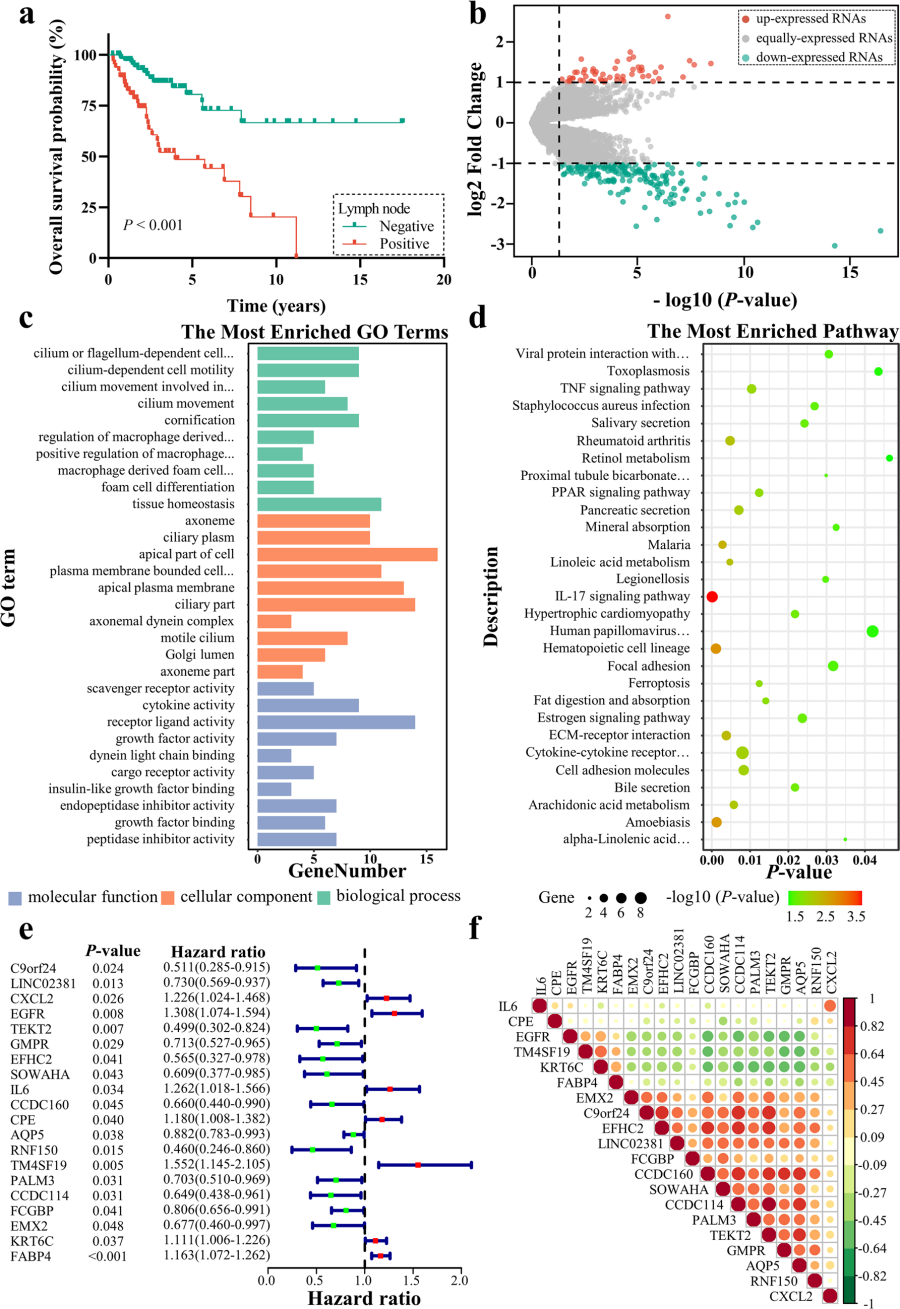
Identification of critical genes associated with LNM. **a** Kaplan–Meier curve for OS based on lymph node status of patients with CCa in the TCGA cohort. **b** The volcano plot shows the DEGs between patients with LNM and without LNM. **c** GO enrichment analysis of the 243 LNM-associated genes. **d** KEGG pathway enrichment analysis of 243 LNM-associated genes. **e** Forest plot of LNM-related genes associated with OS. **f** Pearson correlation analysis was used to determine the correlation among 20 OS-associated genes. The depth of the colour and the size of the circle represent the correlation coefficient between paired genes. Red represents positive correlation and green represents negative correlation

The mRNA expression matrix and clinical information that meet the criteria mentioned above of 178 patients with CCa were obtained from TCGA database. KM survival analysis revealed that patients with lymphatic metastasis had a shorter OS (Fig. 1a). To identify genes associated with LNM, we performed differential expression analysis between CCa patients with positive and negative lymph node. Based on the filter criteria of fold change > 2 and a *P*-value < 0.05, the volcano plot showed there were 243 significant DEGs, including 55 upregulated and 188 downregulated DEGs (Fig. 1b).

### Enrichment analysis of LNM-associated DEGs

To further understand the function and pathway of the 243 identified LNM-associated DEGs, GO and KEGG enrichment analyses were performed. As presented in Fig. 1c, d and Additional files 1 and 2, these LNM-associated genes were mainly enriched in cytokine activity, growth factor activity, cilium-dependent cell motility, cell–cell adhesion via plasma membrane adhesion molecules, cell adhesion molecules, fat digestion and absorption, linoleic acid metabolism and the TNF signalling pathway. The results indicated that these LNM-associated genes may mediate LNM in CCa by regulating the metabolism and motility of cancer cells.

### FABP4 is an independent risk factor for OS in CCa

**Fig. 2:**
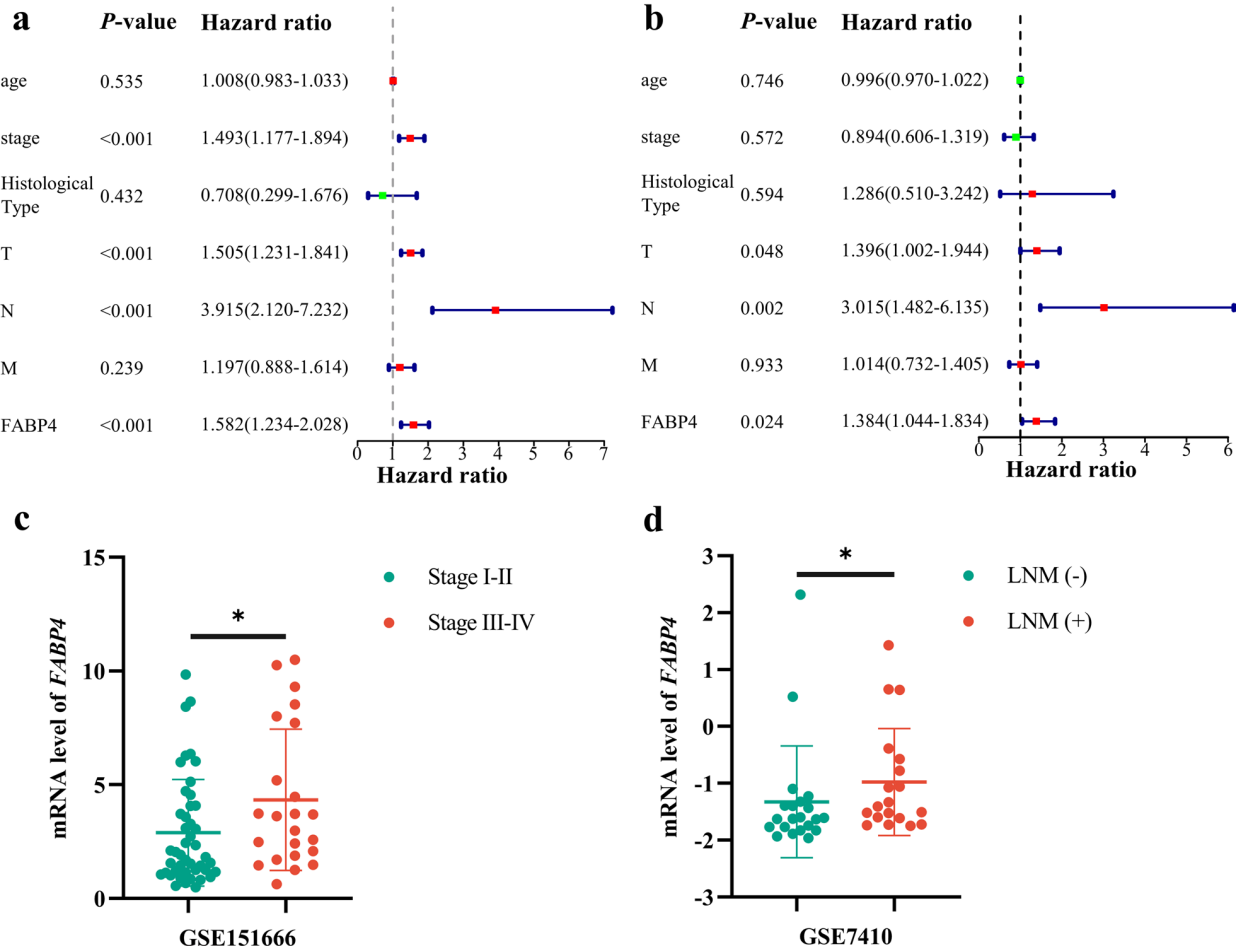
FABP4 is an independent risk factor for LNM in CCa. Forest plots of univariate (**a**) and multivariate (**b**) Cox regression analyses involving FABP4 level and clinical risk factors. The expression of FABP4 in GSE15166 (**c**) and GSE7410 (**d**). *P < 0.05; **P < 0.01; ***P < 0.001; ns. not significant

Univariate Cox regression analysis was performed on 243 LNM-associated DEGs to identify genes significantly associated with OS. The results showed that 20 genes were significantly associated with the OS of CCa patients (Fig. 1e). To assess the correlation among the 20 OS-associated genes, Pearson correlation analysis was performed and revealed that the 20 identified key genes had a co-expression pattern that promoted LNM in the progression of CCa (Fig. 1f). Further survival analysis was performed on the 20 key genes selected above. High expression of EGFR (*P* = 0.014) and FABP4 (*P* = 0.041) and low expression of CCDC160 (*P* = 0.032), EFHC2 (*P* = 0.021), C9orf24 (*P* = 0.009), EMX2 (*P* = 0.017), FCGBP (*P* = 0.048), LINC02381 (*P* = 0.043) and TEKT2 (*P* = 0.002) were associated with worse OS for CCa patients (Additional file 3). Taken together, our studies revealed that FABP4 was highly related to poorer OS in patients with CCa. We then evaluated the independent prognostic force of FABP4. The results from univariate Cox regression analysis showed that FIGO stage (HR = 1.493, *P* < 0.001), T stage (HR = 1.505, *P* < 0.001), lymphatic metastasis (HR = 3.915, *P* < 0.001) and FABP4 (HR = 1.582, *P* < 0.001) had prognostic value for OS in patients with CCa (Fig. 2a). Likewise, multivariate Cox regression analysis showed that T stage (HR = 1.396, *P* = 0.048), lymphatic metastasis (HR = 3.015, *P* = 0.002) and FABP4 (HR = 1.384, *P* = 0.024) were independent prognostic factors for OS in patients with CCa (Fig. 2b).

### FABP4 is an independent risk factor for LNM in CCa

To assess the relationship between FABP4 and LNM, we performed univariate analysis and multivariate analysis in patients with CCa. As shown in Table 3, univariate analysis revealed that FABP4 (*P* = 0.001), M stage (*P* < 0.001), FIGO stage (*P* < 0.001) and T stage (*P* = 0.004) were risk factors for LNM of CCa. Multivariate analysis revealed that FABP4 (*P* = 0.011), M stage (*P* = 0.028) and FIGO stage (*P* < 0.001) were independent risk factors for LNM in CCa.

**Table 3:** Analysis of the risk factors for LNM in patients with CCa (N = 178)

Variables	Univariate regression analyses			Multivariate regression analyses		
	**OR**	**95% CI**	**P**	**OR**	**95% CI**	**P**
FABP4	1.62	1.215–2.160	0.001	1.582	1.110–2.254	0.011
M stage			< 0.001			0.028
M1 vs. M0	17.25	3.549–83.850	< 0.001	5.365	0.595–48.401	0.134
Unknown vs. M0	2.39	1.229–4.645	0.01	2.858	1.257–6.498	0.012
FIGO stage			< 0.001			< 0.001
Stage II vs. Stage I	1.033	0.397–2.687	0.947	0.881	0.318–2.442	0.808
Stage III vs. Stage I	24.792	5.266–116.725	< 0.001	32.671	6.435–165.881	< 0.001
Stage IV vs. Stage I	31.875	6.905–147.132	< 0.001	13.789	2.231–85.230	0.005
Unknown vs. Stage I	–	–	0.999	–		–
T stage			0.004			0.803
T2 vs. T1	1.725	0.798–3.728	0.165	–	–	0.441
T3 vs. T1	13.226	1.485–117.755	0.021	–	–	0.489
T4 vs. T1	4.761	1.480–15.321	0.009	–	–	0.345
Unknown vs. T1	6.613	1.219–35.878	0.029	–	–	0.761
Age	1.013	0.989–1.038	0.297	–	–	–
Histological Type SCC vs. AC	0.566	0.246–1.300	0.18	–	–	–

*CCa* cervical cancer, *FIGO* International Federation of Gynecology and Obstetrics, *SCC* squamous cell carcinoma, *AC* Adenocarcinoma

### External validation of the association between FABP4 and the progression of CCa

**Fig. 3:**
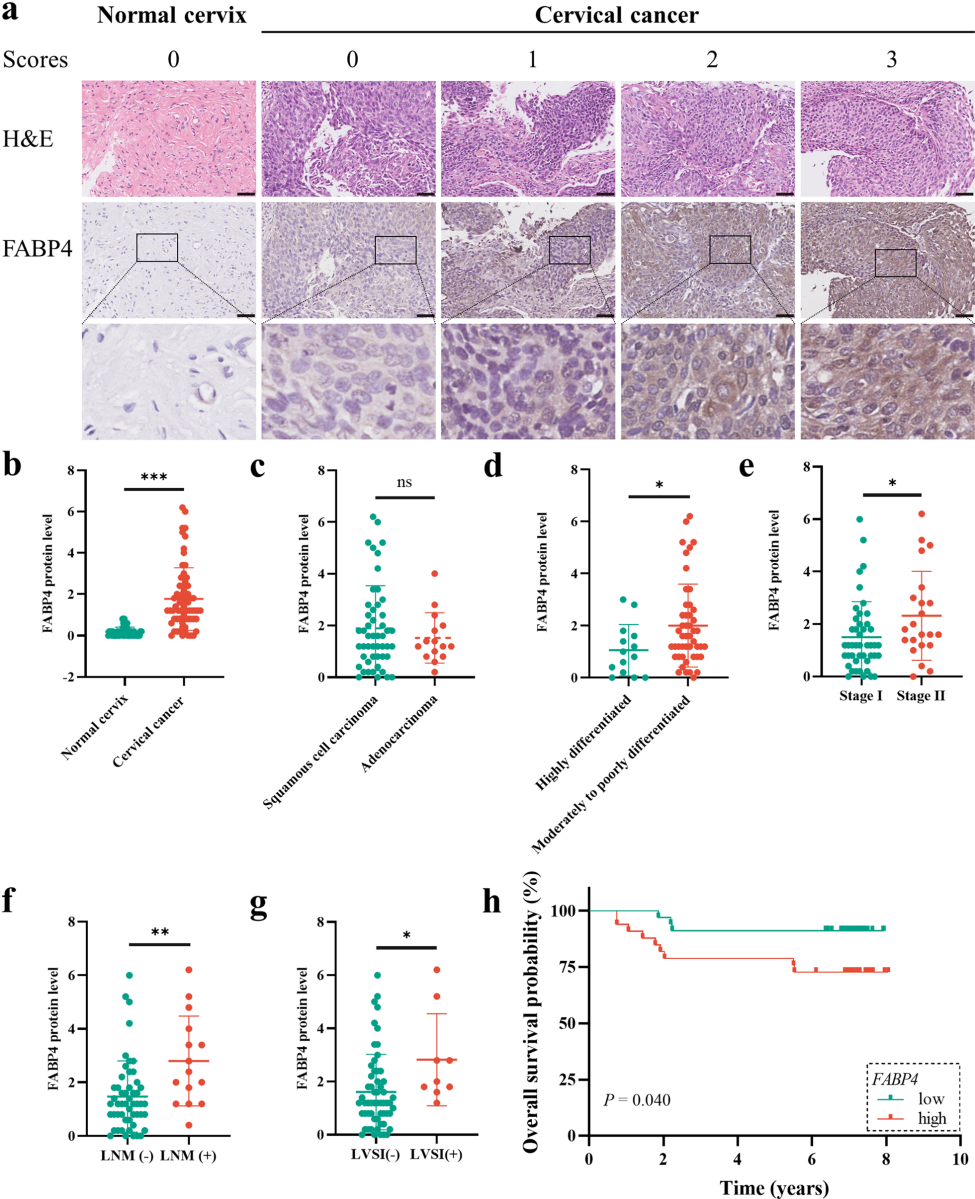
The expression of FABP4 was elevated and correlated with LNM and poorer prognosis in CCa. **a** Representative images of IHC staining of FABP4 in normal cervical and cervical cancer tissues. Scale bar 50 μm. Scatter diagram showing the distribution of FABP4 levels in different disease statuses (**b**), histological types (**c**), grades (**d**), FIGO stages (**e**), lymph node statuses (**f**) and lymphovascular space involvement statuses (**g**). **h** Kaplan–Meier curve of OS for patients in the high FABP4 expression and low FABP4 expression groups. *P < 0.05; **P < 0.01; ***P < 0.001; ns. not significant

To validate the pro-oncogenic effect of FABP4 in other datasets, two GEO datasets (GSE151666 and GSE7410) were used to evaluate the expression of FABP4. As expected, FABP4 was highly expressed in advanced CCa patients (Fig. 2c). Compared with patients without LNM, FABP4 expression was increased in tumour tissues in patients with LNM (Fig. 2d). These results further confirmed the association between FABP4 overexpression and LNM. To investigate the protein expression of FABP4 in cervical tissues, we performed IHC using 48 normal cervical and 67 CCa samples with available clinical data. FABP4 levels were dramatically increased in CCa samples compared with normal cervical samples (Fig. 3a, b). However, there was no difference in the level of FABP4 between squamous cell carcinoma and adenocarcinoma (Fig. 3c). Among all cancer tissues, we found that moderately to poorly differentiated and advanced FIGO stage CCa tissues had higher levels of FABP4 (Fig. 3d, e), demonstrating that FABP4 was associated with CCa progression. In addition, FABP4 was significantly overexpressed in samples with lymphovascular space involvement (LVSI) and LNM (Fig. 3f, g, Additional file 4). Moreover, survival analysis suggested that patients with elevated FABP4 level significantly correlated with poorer OS. The five-year survival rate of FABP4-overexpresing group was 78.79%, and that of group with FABP4-lowexpresing was 91.18% (Fig. 3h). Taken together, the results of IHC confirmed our conclusion from bioinformatics analysis that overexpression of FABP4 promotes LNM and is associated with a shorter OS in CCa.

### Knock down of FABP4 inhibits the proliferation, migration and invasion of cervical cancer cells

**Fig. 4:**
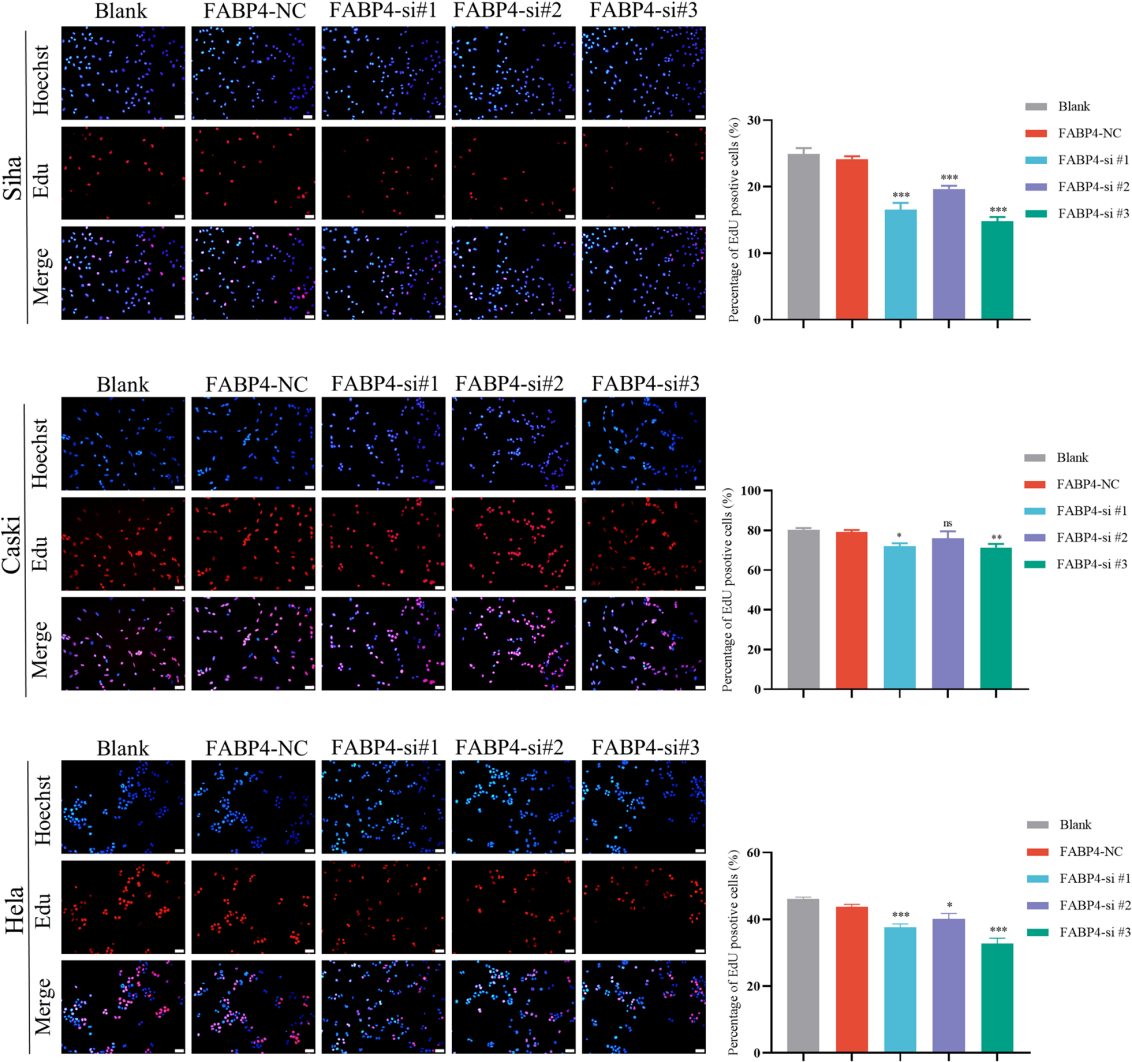
Knock down of FABP4 inhibits the proliferation of cervical cancer cells. Edu assays demonstrated the difference in the proliferative abilities of SiHa, Caski and HeLa cells after knocking down FABP4. Scale bar 50 μm. *P < 0.05; **P < 0.01; ***P < 0.001; ns. not significant

**Fig. 5:**
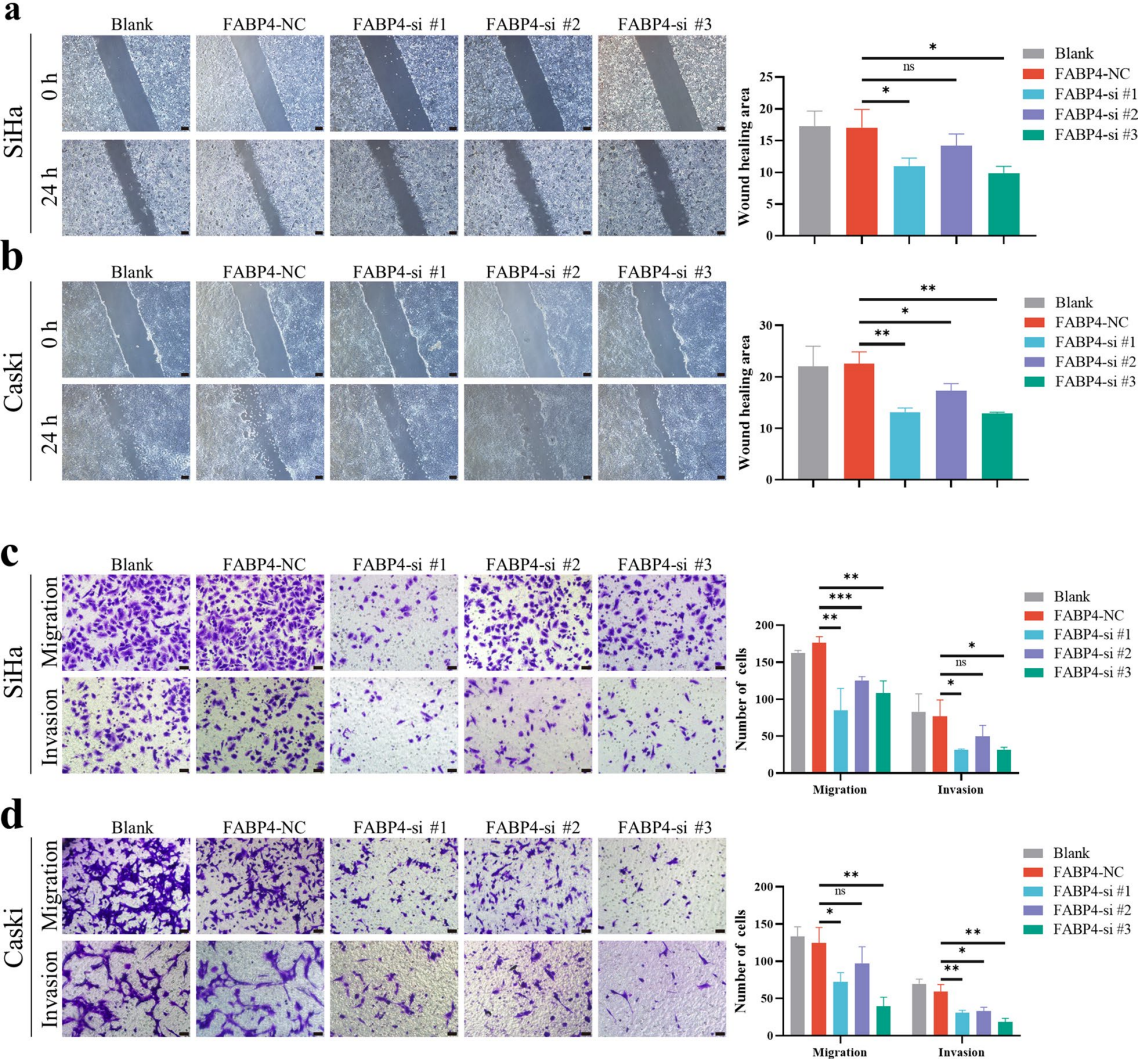
Knock down of FABP4 inhibits the migration and invasion of cervical cancer cells. **a**, **b** Wound healing assay indicated the effects of FABP4 on the migratory abilities of SiHa and Caski cells. Scale bar 200 μm. **c**, **d** Transwell assays demonstrated the difference in the migratory and invasive properties of SiHa and Caski cells after knocking down FABP4. Scale bar 50 μm. *P < 0.05; **P < 0.01; ***P < 0.001; ns. not significant

To investigate the functional role of FABP4 in CCa, the expression of FABP4 was downregulated in HeLa, SiHa and Caski cells using siRNAs. qRT-PCR assay was performed to validate interference efficiency of siRNAs (Additional file 5a). As shown in Fig. 4, Edu assays showed that the proliferative abilities of cervical cancer cells (HeLa, SiHa and Caski) were decreased after knockdown of FABP4. In order to investigate whether FABP4 affects the motility of cervical cancer cells, Wound healing and Transwell assays were performed and demonstrated that FABP4 knockdown led to a significant reduction in both migration and invasion in SiHa and Caski cells but not in HeLa cells (Fig. 5, Additional file 5b, c). These results suggested that FABP4 has certain biological functions in cervical cancer cells.

### FABP4 regulated the process of EMT in CCa via the activation of AKT signalling pathway

**Fig. 6:**
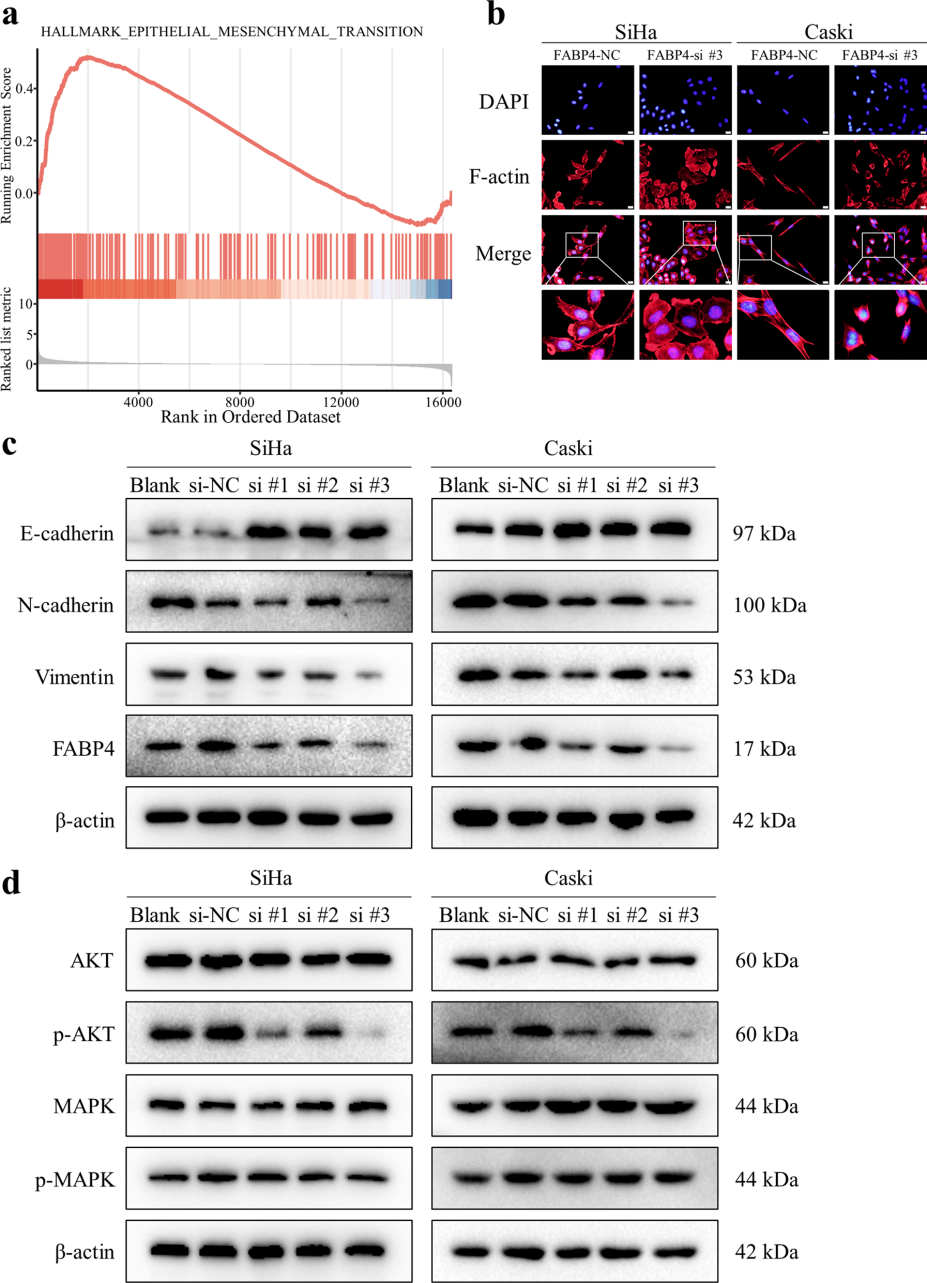
Knock down of FABP4 in cervical cancer cells deactivates the EMT process through AKT pathway. **a** GSEA indicated that EMT was significantly enriched in highly expressed FABP4 samples in TCGA cohort. **b** Morphological changes in cervical cancer cells with downregulated FABP4 were demonstrated by F-actin staining. Scale bar 20 μm. **c** Western blotting analysis of E-cadherin, N-cadherin Vimentin and FABP4 expression in cervical cancer cells after silencing FABP4 expression. **d** Western blotting analysis of AKT, p-AKT, MAPK and p-MAPK expression in cervical cancer cells after silencing FABP4 expression

To elucidate the underlying biological states or processes of FABP4 in CCa, we performed GSEA in TCGA cohort and found that epithelial mesenchymal transition (EMT), IL2-STAT5 signalling, the P53 pathway, KRAS signalling and TGF-β signalling were significantly enriched in the highly expressed FABP4 group and oxidative phosphorylation was significantly enriched in the low-expressed FABP4 group (Fig. 6a and Additional file 6). Likewise, we performed GSEA in GSE15166 and GSE7410 datasets and found that EMT was also significantly enriched in the advanced stage patients and LNM ( +) patients (Additional file 7a). EMT has been confirmed as a key mechanism underlying tumour metastasis [18]. We next investigated whether FABP4 mediated the invasion of CCa through EMT. Cytoskeletal F-actin was stained with phalloidin and was more turbulent after FABP4 knockout (Fig. 6b). At the same time, the slender SiHa and Caski cells became relative round and short (Additional file 7b). These results indicated that FABP4 is involved in cytoskeletal rearrangement. Western blotting results showed that E-cadherin was increased and N-cadherin and Vimentin were decreased after silencing FABP4 expression (Fig. 6c and Additional file 7c). Various studies have shown that AKT and MAPK signalling pathway can affect the EMT process in a variety of ways to influence tumour progression [19, 20]. Then, we evaluated the levels of phosphorylated AKT and phosphorylated MAPK after FABP4 knockdown by western blotting. Our results showed that decreased FABP4 expression inhibits the activation of phosphorylated AKT, but had no effect on MAPK phosphorylation (Fig. 6d and Additional file 7d). These results suggested that FABP4 could promote the EMT process in CCa through the activation of AKT signalling pathway.

## Discussion

In this study, we found that FABP4 is highly associated with LNM and poor prognosis in patients with CCa. This finding is vitally important, as lymph node status has become a crucial clinical issue for the appropriate staging of CCa patients according to the new FIGO staging system. Furthermore, loss of FABP4 suppresses the proliferation, migration and invasion of cervical cancer cells, suggesting that FABP4 has a biological function and is not merely a bystander in CCa. These results revealed that FABP4 could enhance the malignant properties of cervical cancer cells and might serve as a potential biomarker of evaluating lymph node status and postoperative OS in patients with CCa.

Recently, several clinicopathological characteristics have been reported to be risk factors for LNM in CCa, such as advanced stage [21], large tumour volume [22], deep stromal invasion [23], LVSI [24], and high serum squamous cell carcinoma antigens [25]. In the current study, univariate analysis indicated that FIGO stage, T stage, M stage and expression of FABP4 were related to LNM. Multivariate analysis revealed that FIGO stage and FABP4 were independent risk factors for LNM in CCa. We hypothesized that it might be possible to preoperatively evaluate the lymph node status of patients by detecting FABP4 levels in colposcopic biopsy tissue, which provides clinicians with new evidence to determine whether a patient needs surgery or definitive chemoradiotherapy. In addition, we found that overexpression of FABP4 indicated poor OS and that FABP4 could also be used as an independent prognostic factor to assess OS after treatment. These results provide clinical insights for the molecular classification of patients with CCa and suggest that more intensive postoperative treatment or individualized treatment is needed for patients with FABP4-overexpressing CCa.

LNM is a complex process that is precisely regulated by specific genes to enhance the invasive ability of cancer cells to be more aggressive [26]. The process involves several steps, including tumour secretion of lymphangiogenic factors [27–29], tumour lymphangiogenesis [28], lymph node lymphangiogenesis [30], and lymphatic vessel activation of metastatic tumour cells [31]. For example, PTPRM upregulation is relevant to LNM of CCa through VEGF-C-dependent lymphangiogenesis and Src-AKT signalling pathway-mediated EMT [32]. RACK1 promotes the invasive activities and LNM of CCa via galectin 1 [33]. In this study, we identified a set of LNM-associated genes related to the prognosis of CCa. Among these LNM-associated genes, we found that FABP4 was most significantly associated with LNM. Furthermore, we demonstrated that FABP4 serves as a participant, not a bystander, in promoting LNM in CCa through a variety of in vitro assays. After silencing the expression of FABP4 in SiHa and Caski cells, the abilities of migration and invasion were restrained, the expression of an epithelial marker (E-cadherin) was increased, and mesenchymal markers (N-cadherin and Vimentin) were decreased. Nevertheless, we did not observe this phenomenon in HeLa cells. This may be related to the different genetic backgrounds of these cell lines, thus the role of FABP4 in HeLa cells requires further investigation.

Fatty acid-binding proteins that are highly expressed in tissue associated with high metabolic activity or lipid storage are a group of homologous small molecule cytoplasmic proteins that can transport a variety of hydrophobic fatty acids [34, 35]. FABP4 is a member of fatty acid binding protein family and can maintain adipocyte homeostasis and regulate lipolysis and adipogenesis by interacting with hormone-sensitive lipase and peroxisome PPAR-γ, respectively [36]. Various studies showed that FABP4 inhibitors could restrict the progression of several tumours by inhibiting or reducing tumour cell proliferation, metastasis, and drug resistance [16, 37, 38]. Our study has indicated that FABP4 plays an aggressive role in progression of CCa, suggesting that FABP4 inhibitors might be a potential therapeutic strategy for FABP4-overexpressing CCa patients. Nevertheless, Zhong, C. Q. et al. demonstrated that FABP4 low-expression promotes the proliferative and invasive properties of hepatocellular carcinoma cells and predicts short OS and recurrence free survival [39], which contradicts our conclusions. This might be due to the heterogeneity among different cancers, and understanding why FABP4 plays a different role in various cancers requires further research. In recent studies, FABP4 has been shown to promote cancer progression by regulating lipid metabolism [16], the AKT pathway [40] and EMT [14]. In our study, we found that EMT, IL2-STAT5 signalling, the P53 pathway, KRAS signalling and TGF-β signalling were significantly enriched in tissues highly expressing FABP4 by GSEA. Subsequently, we revealed that the cytoskeletons of cervical cancer cells were reprogrammed and that the EMT process and AKT pathway were inactivated after FABP4 inhibition, which provides a new perspective on the role of EMT in LNM.

The association of FABP4 with LNM in cancer patients has been proposed. To date, this is the first study to show the relationship between FABP4 and LNM in CCa. Zhang, Y. et al*.* revealed that the FABP4 protein level in colorectal cancer tissues was increased and correlated with lymphatic metastasis [41]. Luo et al*.* [42] revealed that the percentage of patients with FABP4 protein overexpression was significantly higher in pancreatic ductal adenocarcinoma patients with LNM than in patients without LNM. However, given that these studies were single-centre studies, the conclusions have not been well substantiated. An important advantage of our study is that our conclusion was confirmed in multiple databases and in clinical specimens, which verifies the accuracy and reliability of our results. However, for clinical transformation, more clinical samples need to be included to further confirm our conclusions. At the same time, what promotes the expression of FABP4 and the underlying mechanism of FABP4-mediated LNM in CCa warrants future investigation.

## Conclusions

In conclusion, this study reveals the important role of FABP4 in the LNM and poor prognosis of CCa patients. We speculate that FABP4 is a potential and promising biomarker of evaluating lymph node status and survival as well as a novel therapeutic target for lymph node-positive patients with CCa.

## Supplementary Information

**Additional file 1.** The results of GO enrichment analysis of 243 LNM-associated genes.

**Additional file 2.** The results of KEGG pathway enrichment analysis of 243 LNM-associated genes.

**Additional file 3. **KM survival curves were generated for selected critical genes extracted from the comparison of groups of high (red) and low (green) gene expression.

**Additional file 4. **Representative immunohistochemical images of lymph node negative and lymph node positive patients. Scale bar 50 μm.

**Additional file 5. **(a) qRT-PCR indicated the siRNAs interference efficiency of FABP4. Wound healing (b) and Transwell (c) assays demonstrated the effects of FABP4 on the migratory and invasive properties of HeLa cells. Scale bar of Wound healing 200 μm. Scale bar of Transwell assays 50 μm. *P < 0.05; **P < 0.01; ***P < 0.001; ns. not significant.

**Additional file 6. ** The results of GESA in TCGA cohort.

**Additional file 7. **(a) GSEA indicated that EMT was significantly enriched in the advanced stage patients and lymph node positive patients in GSE15166 and GSE7410. (b) Morphological changes in cervical cancer cells with downregulated FABP4. Scale bar 50μm. (c) Quantitative analysis of EMT-related proteins by western blotting. (d) Quantitative analysis of signalling pathway related proteins by western blotting. *P < 0.05; **P < 0.01; ***P < 0.001; ns. not significant.

## Data Availability

Public datasets of CCa analysed during the current study can be retrieved from TCGA database (available at: https://portal.gdc.cancer.gov/). GSE15166 and GSE7410 datasets can be retrieved from the GEO database (available at: https://www.ncbi.nlm.nih.gov/geo/). Other data that support the findings of this study are available from the corresponding author on reasonable request.

## Abbreviations

LNM: Lymph node metastasis
CCa: Cervical cancer
DEGs: Differentially expressed genes
OS: Overall survival
FABP4: Fatty acid binding protein 4
FIGO: International Federation of Gynecology and Obstetrics
TCGA: The Cancer Genome Atlas
GEO: Gene Expression Omnibus
GO: Gene Oncology
KEGG: Kyoto Encyclopedia of Genes and Genomes
GSEA: Gene set enrichment analysis
IHC: Immunohistochemistry
siRNAs: Small interfering RNAs
KM: Kaplan–Meier
LVSI: Lymphovascular space involvement
EMT: Epithelial mesenchymal transition
