# Supplementary figures and images for: FABP4 is an independent risk factor for lymph node metastasis and poor prognosis in patients with cervical cancer
Source: Cancer Cell Int. 2021 Oct 26;21:568. doi: 10.1186/s12935-021-02273-4 (PMC8549317; doi:10.1186/s12935-021-02273-4)

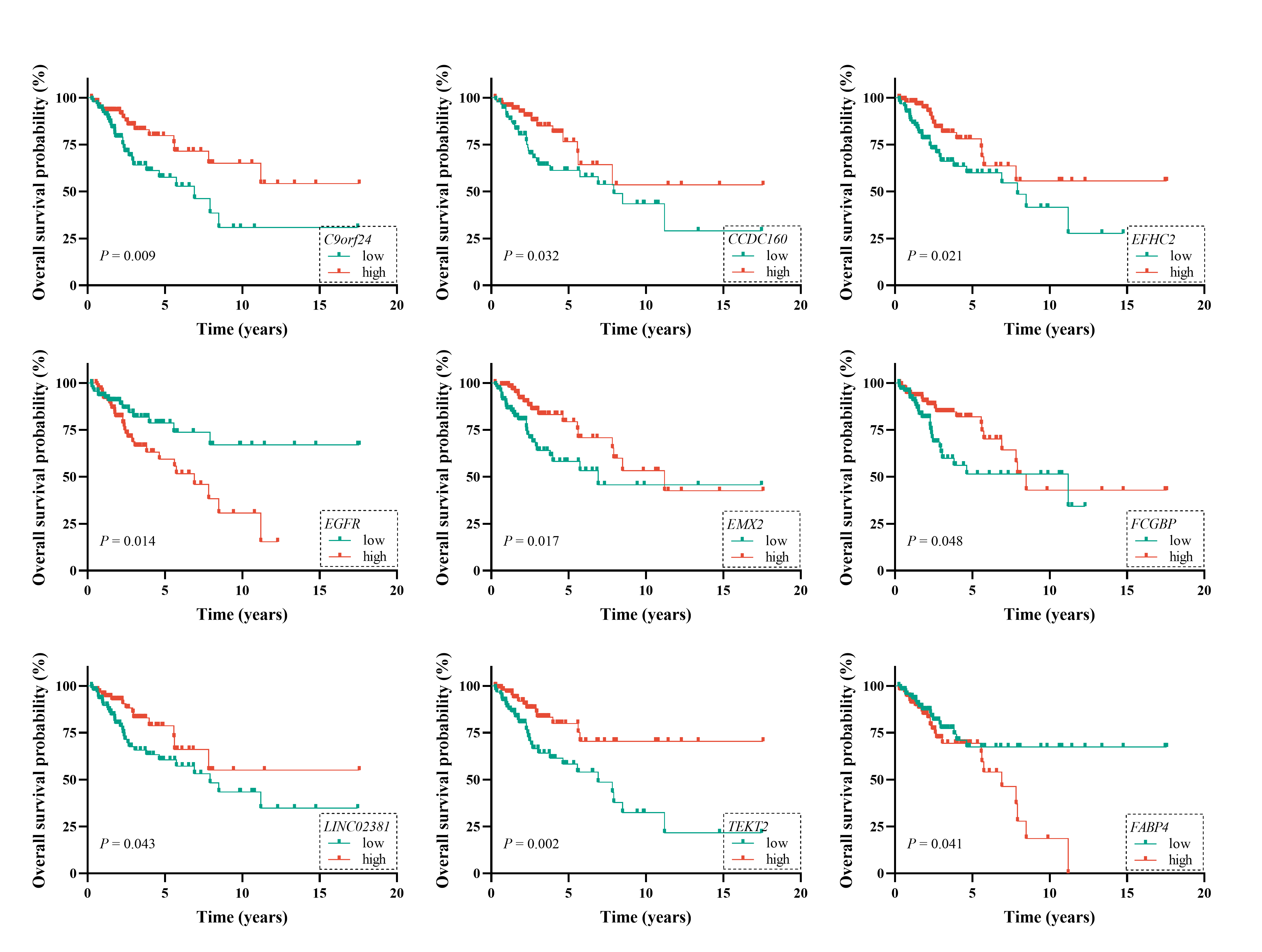

Supplement: Supplementary file 3 — Additional file 3. KM survival curves were generated for selected critical genes extracted from the comparison of groups of high (red) and low (green) gene expression. [file 12935_2021_2273_MOESM3_ESM.tif]

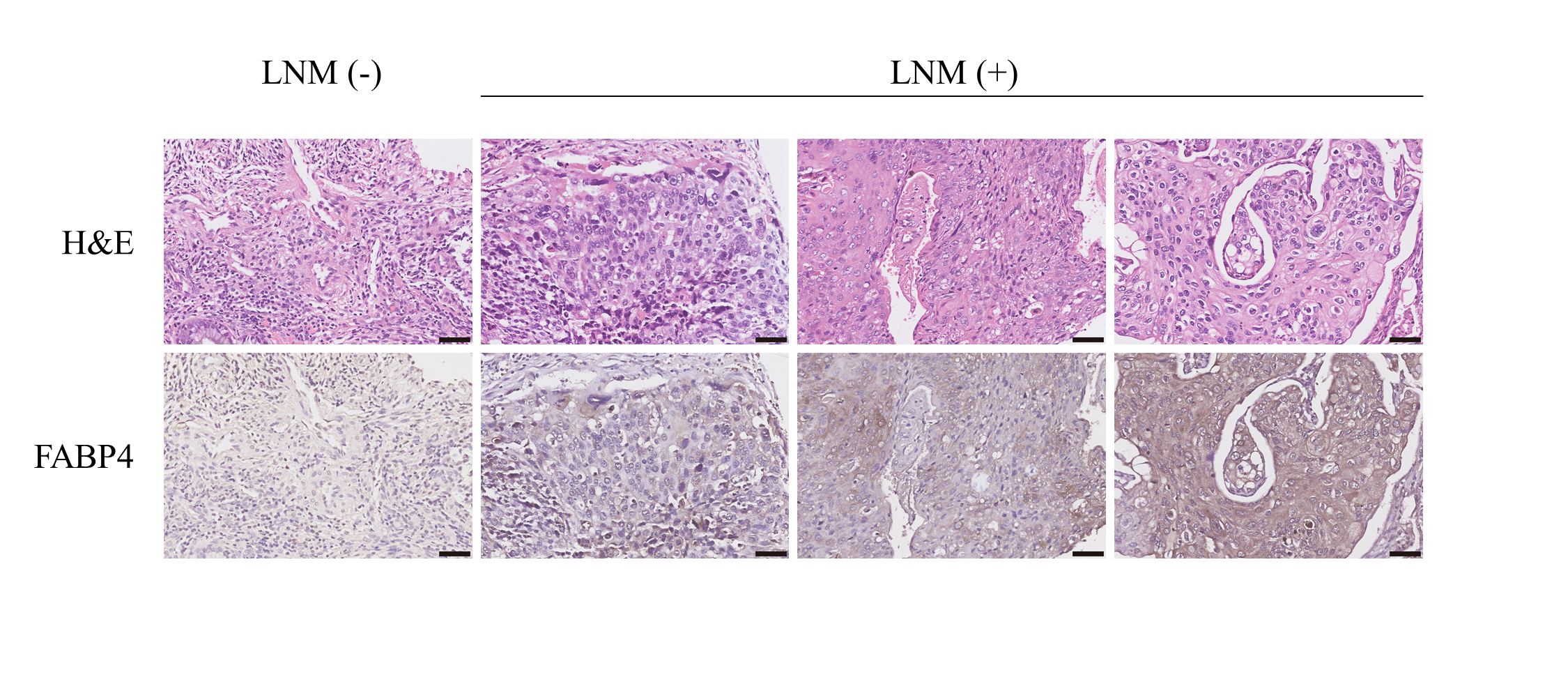

Supplement: Supplementary file 4 — Additional file 4. Representative immunohistochemical images of lymph node negative and lymph node positive patients. Scale bar 50 μm. [file 12935_2021_2273_MOESM4_ESM.tif]

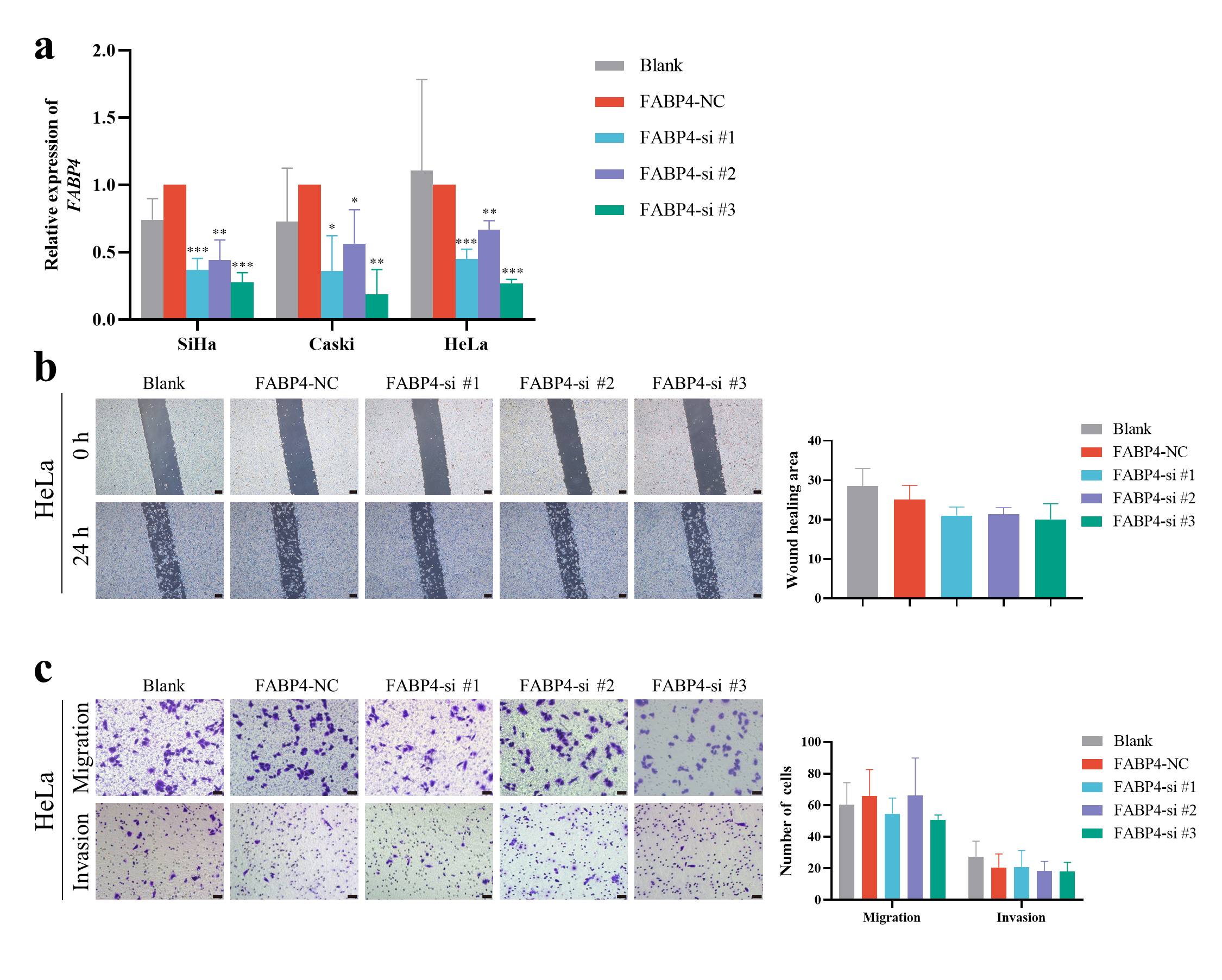

Supplement: Supplementary file 5 — Additional file 5. (a) qRT-PCR indicated the siRNAs interference efficiency of FABP4. Wound healing (b) and Transwell (c) assays demonstrated the effects of FABP4 on the migratory and invasive properties of HeLa cells. Scale bar of Wound healing 200 μm. Scale bar of Transwell assays 50 μm. *P < 0.05; **P < 0.01; ***P < 0.001; ns. not significant. [file 12935_2021_2273_MOESM5_ESM.tif]

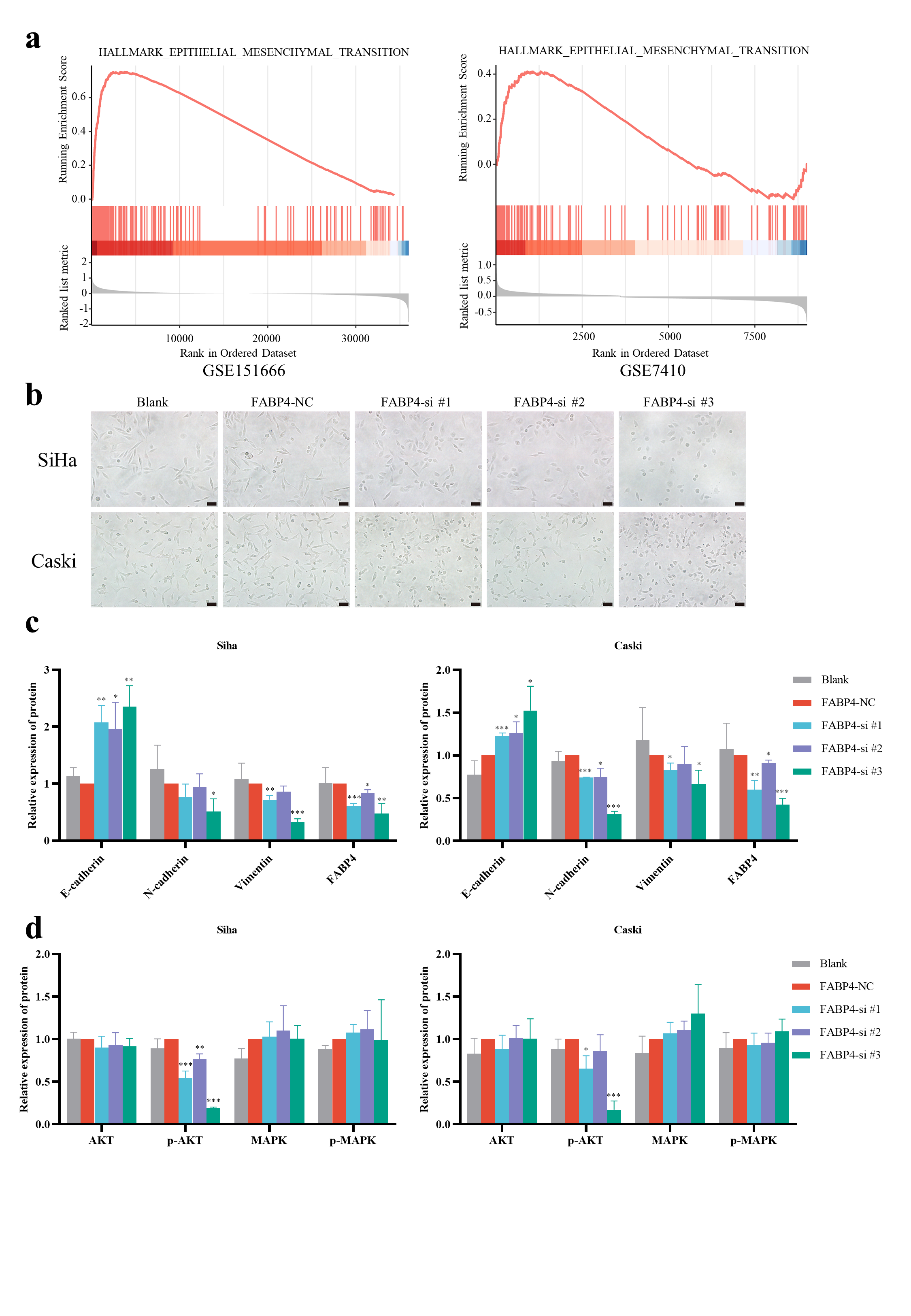

Supplement: Supplementary file 7 — Additional file 7. (a) GSEA indicated that EMT was significantly enriched in the advanced stage patients and lymph node positive patients in GSE15166 and GSE7410. (b) Morphological changes in cervical cancer cells with downregulated FABP4. Scale bar 50μm. (c) Quantitative analysis of EMT-related proteins by western blotting. (d) Quantitative analysis of signalling pathway related proteins by western blotting. *P < 0.05; **P < 0.01; ***P < 0.001; ns. not significant. [file 12935_2021_2273_MOESM7_ESM.tif]
